# Definition of Linear Color Models in the RGB Vector Color Space to Detect Red Peaches in Orchard Images Taken under Natural Illumination

**DOI:** 10.3390/s120607701

**Published:** 2012-06-07

**Authors:** Mercè Teixidó, Davinia Font, Tomàs Pallejà, Marcel Tresanchez, Miquel Nogués, Jordi Palacín

**Affiliations:** Department of Computer Science and Industrial Engineering, University of Lleida, Jaume II, 69, 25001 Lleida, Spain; E-Mails: mteixido@diei.udl.cat (M.T.); dfont@diei.udl.cat (D.F.); tpalleja@diei.udl.cat (T.P.); mtresanchez@diei.udl.cat (M.T.); mnogues@diei.udl.cat (M.N.)

**Keywords:** red-peach harvesting, fruit detection, RGB color space

## Abstract

This work proposes the detection of red peaches in orchard images based on the definition of different linear color models in the RGB vector color space. The classification and segmentation of the pixels of the image is then performed by comparing the color distance from each pixel to the different previously defined linear color models. The methodology proposed has been tested with images obtained in a real orchard under natural light. The peach variety in the orchard was the paraguayo (*Prunus persica* var. platycarpa) peach with red skin. The segmentation results showed that the area of the red peaches in the images was detected with an average error of 11.6%; 19.7% in the case of bright illumination; 8.2% in the case of low illumination; 8.6% for occlusion up to 33%; 12.2% in the case of occlusion between 34 and 66%; and 23% for occlusion above 66%. Finally, a methodology was proposed to estimate the diameter of the fruits based on an ellipsoidal fitting. A first diameter was obtained by using all the contour pixels and a second diameter was obtained by rejecting some pixels of the contour. This approach enables a rough estimate of the fruit occlusion percentage range by comparing the two diameter estimates.

## 1. Introduction

Automatic fruit detection in orchards has many useful applications, such as estimating the expected yield, optimizing the application of agrochemicals, estimating the required harvesting time and storage area after harvesting, and even automatic fruit picking. In all cases an accurate estimate of the size and number of fruit, something that is currently based on experience or on a manual analysis of a limited area of the orchard, is very interesting for the growers.

This work proposes a methodology for automatic fruit detection in RGB color images of orchards containing red peaches. The main goal of the proposed methodology is to detect individual fruit and a secondary goal is to estimate the size of the fruit. In future works this information will be used to guide robotized devices in the orchard to pick individual fruit from the trees automatically. The development of automatic harvesting has many practical advantages, such as reducing the stress on the fruit, the time needed and the cost of harvesting (currently 50% of the overall production cost in developed countries), and optimizing the number of harvesting passes in order to maximize the market value of the overall fruit production.

Nowadays, image-processing techniques are used mainly as a non-intrusive technique [1,2] to estimate fruit quality [3–5]. For example, in [6–8] different fruit categories were defined from detected surface defects and in [9,10] such fruit features as size and color of citrus [9], peaches, apples [10] were analyzed. In [11], there is a summary of the methodologies used to locate fruit on a tree with such applications as yield estimate [12] and automated harvesting of citrus [13,14], apples [15–17], cherries [18], oranges [19,20], peaches [21], *etc*. The strategies summarized in [11] to detect fruit on trees were based on intensity, spectral and range images. Intensity and spectral images are ideal when the fruit and the background are different, but can be strongly affected by shadows and confusing regions.

The contribution of this work is the proposal to apply a new methodology to detect red peaches in images of orchards. The methodology is based on the manual definition of different linear color models in the RGB vector color space without any transformation [22]. Then, the classification and segmentation of the individual pixels of the image is performed by comparing the color intensity distance of each pixel from the different linear color models defined previously. A similar empirical methodology performed by dividing the RGB color space into cylinders instead of lines was proposed in [23] to detect a color chart, and in [24] to detect hand skin colors. In [25] the linear color relationship was used to train a linear attractor to detect skin. Other related works such as [26] propose the detection of the green background of a billiard system by defining 20 small color regions in the RGB color space instead of taking advance of the linear relationship defined by the pixels of the objects.

Finally, the fruit segmentation results obtained in this work will be compared with manual fruit labeling to estimate the errors induced in the segmentation process. At this moment, the measurement of the linear color models used to classify the pixels in the image must be based on human experience but the simplicity and specificity of the linear color models used in this work to detect red peaches suggest a future automatic determination of the linear color models based on the analysis of some sample representative images [27].

**Figure 1.**
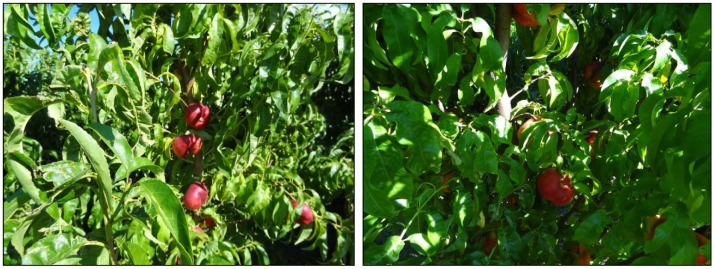
Different lighting conditions in the images from the orchard.

This work deals with images obtained in a real orchard with natural light. Therefore, the fruit may be partially hidden by leaves or branches in the images and may have different lighting and color intensity, depending on the shading and direct incidence of sunlight, thus making classification more difficult. Figure 1 illustrates these effects with two sample images taken under different lighting conditions in the same orchard.

## 2. Area of Application

**Figure 2.**
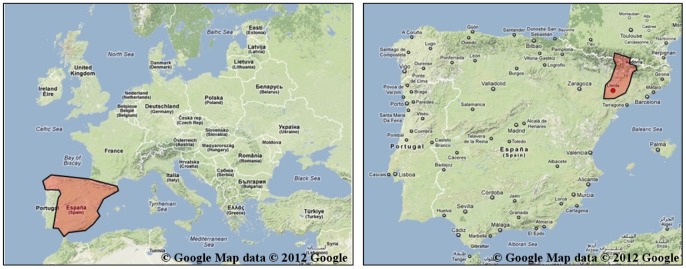
Location of Lleida within Spain (Courtesy of Google).

The orchard images analyzed in this work were obtained in Aitona (41°29′45.23″N, 0°27′35.70″E), a typical agricultural community in the province of Lleida in Northeast Spain (Figure 2). All the images were taken on 24th July 2011 (summer season) at 5:00 pm on a sunny day and in natural daylight. The weather conditions were: temperature of 28 °C, atmospheric pressure of 16.5 hPa, average relative humidity of 55%, and wind speed of 0 Km/h [28]. Due to its favorable climatic conditions, Lleida is one of Europe's leading peach-producing regions, with a production of 401,980 tones in 2011 [29].

The peaches grown in the selected orchard were a paraguayo variety that is very typical in this area. Its main characteristics are: a circular and flattened shape, a red skin when ripe and green when unripe and white flesh. The harvest of this variety starts at the end of May and lasts until late September. The selected orchard was prepared for the production of high-quality fresh fruit and the peaches were in the optimum period for harvesting, with a characteristic red color, and the trees heavily laden with leaves with a characteristic green color.

Figure 1 shows some samples of images acquired in the orchard. These were taken with a commercial Cybershot HX9 SONY camera, with a resolution of 2,592 × 1,944 square pixels, with 24 bits per pixel in the RGB color plane, without applying optical or digital zoom (covering a field of view of 70.7°), and with fully automatic exposure conditions to adapt the dynamic range of the camera to the illumination conditions. The camera was originally placed in the orchard parallel to a row of trees at a fixed height from the ground (1,400 mm) and a fixed distance from the trees (1,200 mm). The camera was moved along the row of trees to simulate the movement of a typical agricultural machine and new images were taken at intervals of 100 mm.

## 3. Approach

### 3.1. Classification Method

The classification method proposed in this work is to use the proximity to some linear color models (or lines) defined in the RGB vector color space to detect the red peaches in the images from the orchard. This specific proposal was originated from the observation that the RGB color distribution of small regions of peaches and leaves follow a linear evolution when represented in a RGB vector color space because small changes in the illumination modifies the pixel color description of the objects. This effect has been reported widely in the literature [27,30] and the proposal of this work is to use it as a base for the segmentation of the images of the orchard.

**Figure 3.**
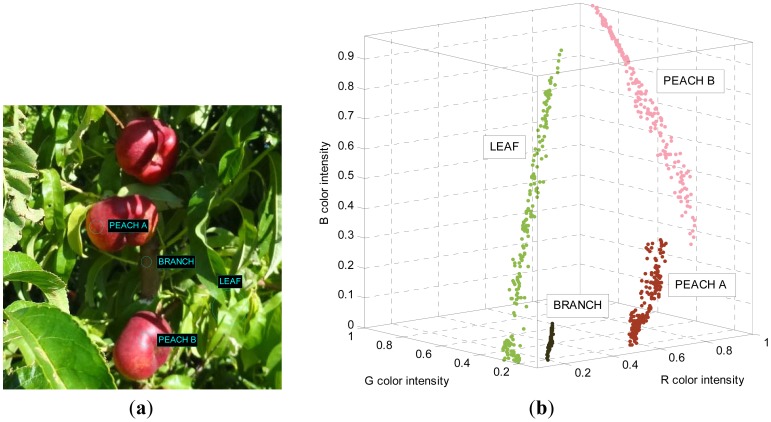
Illustrative image of the orchard with several areas selected (**a**) and representation of the RGB color intensity distribution of the pixels selected in the RGB vector color space (**b**).

**Figure 4.**
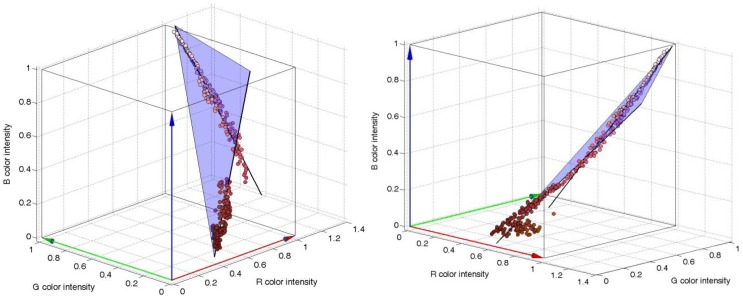
Two additional views of the PEACH A and B areas (Figure 3) plotted with a regression line and an auxiliary plane plotted to illustrate the relationship of both regression lines.

To illustrate this linear relationship, Figure 3(a) shows an image of the orchard with several areas selected and Figure 3(b) shows the RGB color intensities of the selected pixels plotted in the RGB vector color space. The small areas selected in the image in Figure 3 are labeled as: PEACH A; red skin of a paraguayo, PEACH B; red skin of a paraguayo brightly lit by the sun, LEAF; green leaves brightly lit by sunlight, BRANCH; the poorly-lit brown branch of the tree. The observation behind this work is that the RGB color intensities of the pixels corresponding to small areas of peaches, leaves, and other objects plotted in the RGB vector color space follow a linear trajectory that can be modeled by a linear three-dimensional regression [31] that can be interpreted as a linear color model. Following this observation, a unique object can be modeled with different linear trajectories in the RGB vector color space depending on the illumination. For example, the area labeled as PEACH B in Figure 3 seems to be a saturated projection over the plane R = 1 of the regression line defined by PEACH A. Therefore, the class or object PEACH may require the definition of different linear color models to cover all the color and illumination variations of the object in the images. Figure 4 shows two additional views of the PEACH A and B selections that includes their linear regressions and an auxiliary plane to illustrate that both regression lines are very close to define a plane in the RGB vector color space.

The determination of the linear color models in the RGB vector color space of the different objects of interest was performed by selecting a representative number of pixels of each object and by applying a linear least squares to their color intensities in order to obtain a representative three-dimensional line (or linear regression). Once all the objects of interest are defined as linear color models then any image pixel can be classified by computing the Euclidean distance defined from its RGB pixel intensity to the different linear color models available and labeling the pixel with the class name with minor distance. This linear relationship is partially lost when normalizing or transforming the image into other color models, such as the HSI [32]. Finally, the proposed approach assumes implicitly that the decision surfaces between the different linear color models are equidistant so the segmentation can be performed without defining additional threshold levels [33], radial linear proximities [23], segmentation planes [34], or decision surfaces [27].

### 3.2. Empirical Determination of the Linear Color Models

At this moment the proposed classification method has one drawback; the selection of the small area of the image that defines a linear color model has to be performed empirically, requiring a manual experimented operation in a set of representative images from the orchard. Nevertheless, after initial training, this manual selection becomes simple as it can be performed iteratively by selecting different small regions of known objects, plotting its color intensities in a RGB vector color space (see Figure 3(a)), applying a linear three-dimensional regression in each selected region to define preliminary linear color models, and labeling all the pixels of an orchard image according the distance of its R, G, and B color intensities to the defined linear color models. This iterative process will end with definitive linear color models when the contour of the red peaches appears clearly in the labeled image.

At this moment, efforts focused on the automatic determination of the linear color models required to classify the red peaches of the orchard images have had no success because the problem is that a real image contains hundreds of small areas whose pixel color intensities have a linear relationship in the RGB vector color space. Then, the selection of the best linear color model candidates for peach detection still requires manual expert operation in order to select the appropriate and minimum number of linear color models and to increase the speed of the detection procedure.

**Figure 5.**
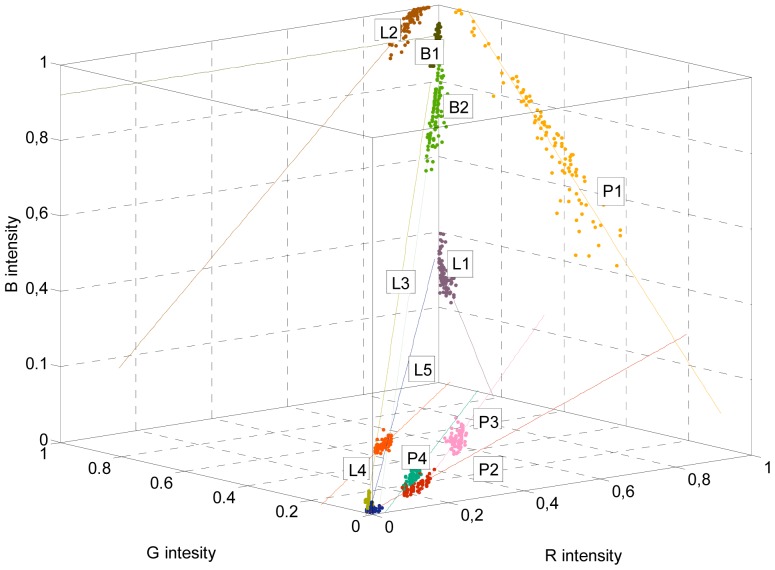
Linear color models proposed to detect red paraguayo peaches.

Finally, Figure 5 and Table 1 show the different linear color models empirically proposed to detect red paraguayo peaches in the orchard images analyzed in this work. Table 1 summarizes the different objects (or class names) and describe briefly the motivation behind each linear color model used. These linear color models do not try to classify all the objects in the image as they are focused on discriminating between red peaches and surrounding objects. Therefore, the proposed linear color models try to describe the different color behavior of red peaches and nearby leaves and branches under different lighting conditions. Other objects that may appear in the images, such as blue sky, are not modeled for this particular application because their linear color model is very close to the color evolution of a whitish leaf (Table 1-L2) and its correct classification is of no practical interest.

### 3.3. Fruit Segmentation

The final segmentation of the fruit in the images is as folows: if a pixel is classified with any “Peach” class the pixel is then labeled as “1” or otherwise “0”. An accurate and representative shape of the fruit is required after segmentation to allow the area and diameter to be estimated. Then, any isolated noisy pixels are removed from the image by deleting associations of pixels with less than 50 elements and the holes in the remaining objects are filled by conventional image processing operations. The proposed methodology is evaluated by comparing the automatic segmentation results with a manual segmentation of the individual paraguayo fruit pieces in the images. Although slow and tedious, this manual procedure has the advantage that the apparent shape of the fruit can be labeled regardless of occlusion by leaves and branches and then the estimate of the percentage of occlusion can be used to develop additional analyses in the future. However, in practice, it is very difficult to mark the contour of the flattened paraguayo peaches precisely in high-resolution images and a difference of less than 3% in the area of the fruit must be considered normal and illustrative of the difficulty of the automatic classification process.

### 3.4. Detecting Individual Fruit Pieces

**Figure 6.**
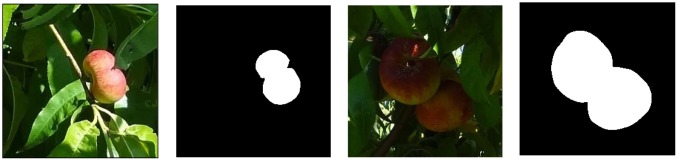
Sample original and segmented images of one (**left**) and two pieces of fruit (**right**).

The detection of individual paraguayo pieces is a very complex problem that is far from being the main aim of this work because of their flattened shape and other specific associated problems, such as occlusion and fruit overlapping. In the case of spherical fruits like oranges [20] and apples, a small part of the contour can be used to estimate the centre and radius of the piece [12], but this methodology is not applicable in the case of paraguayo peaches because of their flattened shape. To illustrate this problem, Figure 6 shows sample images containing one and two paraguayos with a very similar segmented shape. In practice it is very difficult to differentiate overlapping pieces if other information, such as the depth of image, is unavailable. However, in this case, the orchard was heavily pruned to maximize the size and quality of the individual fruit and thus their fresh market value. As a consequence, less than 80% of the individual paraguayo pieces overlap in the images acquired and this overlapping tends to disappear when the camera changes the relative point of view of the trees analyzed [35]. In the future, new efforts will be focused on differentiating between overlapping paraguayo pieces in a less specialized orchard but the assumption in this work is that the fruit do not overlap in the images analyzed.

### 3.5. Peach Size Estimate

**Figure 7.**
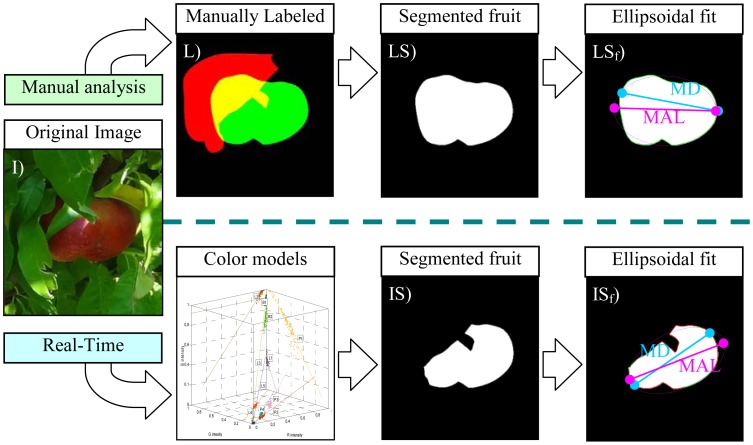
Peach size estimate analysis: (**I**) original sample image, (**L**) manually labeled image, (**LS**) complete fruit segmentation, (**IS**) automatic fruit segmentation (with occlusion), and (**LS_f_**) and (**IS_f_**) results of the diameter estimate.

Estimating the diameter of a segmented piece of fruit has many useful applications, such as selecting the appropriate candidates for automatic fruit picking. In the case of non-occluded paraguayo fruit, the diameter can be easily estimated as the maximum distance between contour pixels, but this value has no sense in the case of partially occluded fruits. The alternative proposal in this work is to model the flattened contour of paraguayo peaches by using an ellipsoidal function whose parameters (location of the two centers and radius) are obtained by applying a non-linear least squares fit [36] with the coordinates defined by the contour pixels of the segmented piece of fruit. This approach has the main advantage that it is a robust standard procedure [37] and after the initial fitting, the similarity between the contour of the fruit and the contour of the fitted ellipse can be evaluated to reject some pixels of the contour and perform a second ellipsoidal fit. In this work, the specific criterion applied to reject the problematic pixels of the contour was the application of a threshold level to the residual of the non-linear least squares fit, a value that was set to 0.15 after a trial and error procedure with synthetic ellipsoidal images. The differences between the two ellipses obtained in this procedure will evidence the effect of the occlusion in the fruit, an aspect that will be analyzed in detail in the next sections. Figure 7 summarizes the analysis performed to compare the peach size estimate obtained with the proposed automatic fruit detection procedure with manual results.

Figure 7-I shows an example real image with a partially occluded red peach that will be used in this description. The manual procedure consists as follows: (1) Figure 7-L shows the results of a manual labeling procedure performed to manually select the complete contour of the fruit in the image (whose area is represented in green color) and the partial contour of the main occluding leaves (whose area is represented in red color), the occluded area of the fruit area appears automatically in a combined color representation (yellow color). The results of this manual fruit selection will be assumed as a perfect representation of the contour of the fruit and the overlapping areas but it is obvious that this manual selection depends largely on the previous visual experience of the human operator with paraguayo peaches as it has to imagine the shape and contour of the fruit in the occluding areas. (2) Figure 7-LS shows the complete fruit segmentation obtained from the previous labeling, but this segmentation can be limited to the non-occluded part of the fruit. (3) Figure 7-LS_f_ shows the results of the two step ellipsoidal fit proposed. In this case all the contour pixels (marked with green dots) of the fruit were used in the second ellipsoidal fit whose shape is plotted with a magenta line. Additionally, the maximum distance (MD, blue line) between contour pixels and the major axis length (MAL, magenta line) of the ellipse are also plotted as a reference for later analysis.

The automatic detection procedure consists as follows: (1) Figure 7-IS shows the real fruit segmentation obtained from the previously defined linear color models. (2) Figure 7-IS_f_ shows the results of the two step ellipsoidal fit proposed. In this case part of the contour pixels (marked with red dots) were discarded in the second ellipsoidal fit whose shape is also plotted with a magenta line. Again, the maximum distance (MD, blue line) of the fruit and the major axis length (MAL, magenta line) of the ellipse are also plotted.

In a real-time application only the segmented image obtained by applying the proposed linear color models will be available but the manual analysis enables the estimate of the error obtained in the determination of the peach size and even the area of the segmented peach.

Table 2 summarizes the different diameter estimate values obtained from the example image analyzed in Figure 7. In an ideal non occluded fruit case (Figure 7-image LS), the value of the diameter coincides with the maximum distance (MD) between pixels computed from the locations of the contour pixels of the fruit. However, the effect of fruit occlusion (Figure 7-image IS) affects strongly the MD value, 5% less in the image analyzed with a very small occlusion. Alternatively, the diameter estimate obtained as the major axis length (MAL) of the resulting two steps ellipsoidal fit is less prone to occlusion; in the case shown in Figure 7 the difference between MAL obtained from the manual and automatic segmented image was only 0.8%.

The estimate of the diameter can be expressed directly in millimeters if an estimate of the distance between the camera and the fruit is available by using a complementary LIDAR sensor [38–40], by using a second camera to create a stereo image to estimate the image depth [41,42], or by taking advantage of the continuous displacement of the camera to combine images with different perspectives of the fruit [43,44].

## 4. Results

Under ideal lighting conditions, ripe paraguayo peaches have a characteristic red color but these conditions are rarely present in orchard images recorded under natural light and peaches can appear partially influenced by shadows, with the colors saturated by bright illumination, and partially occluded. The methodology proposed to detect red peaches in orchard images was evaluated by dividing the set of images of the orchard by different illumination conditions and occlusion percentages because it is expected that the quality of the detection will depend largely on these uncontrolled parameters. The linear color models are proposed to detect ripe red peaches and they must be expected to have problems with unripe peaches with green skin (similar to the leaves of the trees).

**Figure 8.**
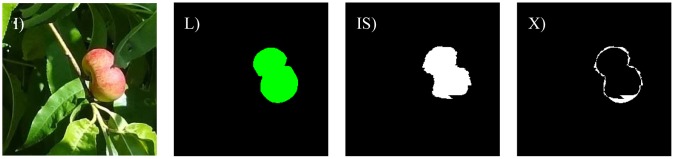
Example peach image results with bright illumination: (**I**) original image, (**L**) manually-labeled fruit, (**IS**) automatic segmentation, and (**X**) pixel differences between (**LS**) and (**IS**) images.

**Figure 9.**
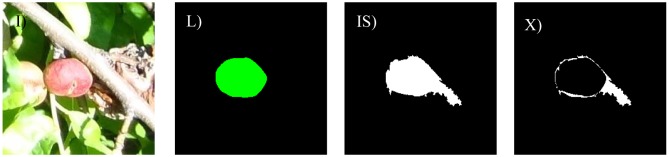
Worst bright illumination image case analyzed.

**Figure 10.**
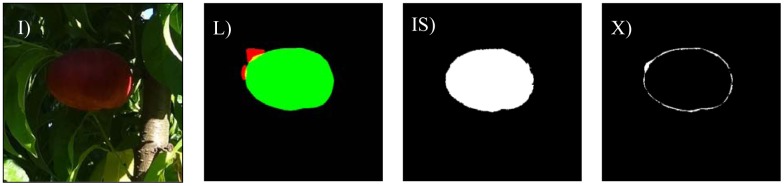
Sample image with low illumination.

**Figure 11.**
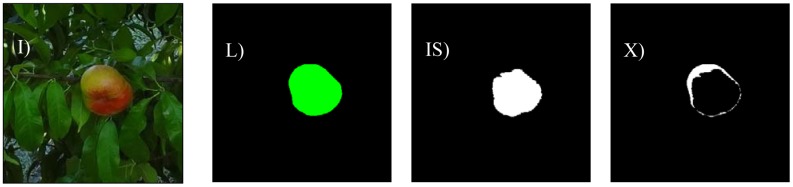
Worst low illumination case.

**Figure 12.**
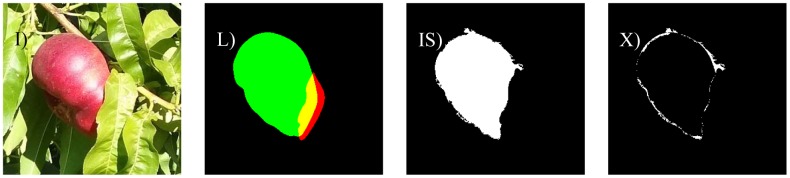
Sample image with bright illumination and occlusion of 9.5%.

**Figure 13.**
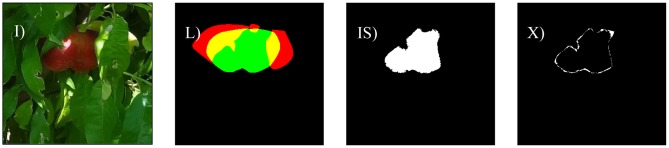
Sample image with an occlusion of 34.5%.

**Figure 14.**
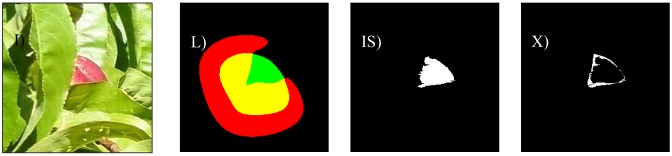
Sample bright illumination image with occlusion of 77.9%.

The different cases considered in this work are: bright illumination, low illumination, occlusion ratios below 33%, between 33 and 66%, and over 66%. In all cases, the difference in terms of area estimate and diameter estimate obtained from the automatic and manual segmentation of the fruit are compared. The following Figures 8–14 will show the results obtained for a representative image of each case considered where (I) is used to mark the original image analyzed; (L) the result of the manual labeling of the complete fruit without occlusion; (IS) the automatically-segmented image with the proposed linear color models; and (X) the pixel differences between the automatically segmented image and the manual selection of the non-occluded part of the labeled fruit. Additionally, Tables 3–7 will summarize the relative differences in the diameter estimates. The symbols of the table are MD to indicate the maximum distance between the contour pixels of the segmented image; MAL-F1 to indicate the major axis length obtained after the first linear least squares fit; MAL-F2 to indicate the major axis length obtained after the second linear least squares fit. In all cases, the diameter value used as a reference was obtained as the maximum distance (MD) between contour pixels computed in the manually labeled image (see Figure 7-LS for reference). The value of the relative differences does not follow a common probability distribution so only the minimum, average, and maximum values are indicated in the tables.

### 4.1. Bright Illumination

Excessive illumination is one of the major problems for any color based detection algorithm, as it tends to saturate the dynamic range of the camera and deteriorate all the color information in the images. Figure 8 shows a representative sample image of this particular case. In a general analysis of non-occluded fruit pieces under bright illumination, the average error of the number of different pixels between the manual and automatic segmentation was 19.7% with a minimum and maximum value of 4.7% and 34.8%, respectively. Figure 9 shows the images corresponding to the worst-classification case under bright illumination where a dead leaf was mistaken for part of a peach because of its saturated brown color.

Table 3 summarizes the relative error in the estimate of the diameter of the fruit relative to the MD value obtained in the manually labeled and segmented image. In the case of a real segmented image (Figure 8-IS) the MD value has changed the 6.4% whereas the MAL results obtained in the first (F1) and second exponential fit (F2) applied to the automatically segmented image have almost no variations with an average error lower than 3.6%. Therefore, in this case the proposed MAL diameter estimate has better results in average.

### 4.2. Low Illumination

Low lighting is another of the major problems when applying a color-based detection algorithm as the color information blurs in dark images. Figure 10 shows a representative sample image of a poorly-illuminated paraguayo peach. In a general analysis, the average error in the number of different pixels between the manual and automatic segmentation was 8.2% with a minimum and maximum value of 4.2% and 12.3% respectively. Figure 11 shows the images corresponding to the worst classification case under low illumination where the upper part of an unripe peach was mistaken for part of a leaf because of its dark green color.

Table 4 summarizes the average relative error in the estimate of the diameter of the fruit. In the case of a real segmented image (Figure 10-IS) the MD value has changed the 6.2% whereas the MAL results obtained in the first (F1) and second exponential fit (F2) applied to the automatically segmented image have changed the 5.6% and 8.0% respectively. Therefore, in this case there are little differences between the MD and MAL diameter estimate.

### 4.3. Peaches with Occlusion Ratios Lower than 33%

Regardless of the level of lighting, occlusion is the major problem when estimating the diameter of a fruit. Figure 12 shows a sample image of a paraguayo peach with an occlusion of 9.5%. In a general analysis of partially occluded (lower than 33%) fruit pieces, the average error in the number of different pixels between a manually-labeled and automatically-segmented image was 8.6% with minimum and maximum values of 2.9% and 19.8% respectively.

Table 5 summarizes the average relative error in the estimate of the diameter of the fruit. In the case of a real segmented image (Figure 12-IS) the MD and MAL-F1 relative error was very similar (5.8% and 5.6% respectively) whereas the second exponential fit (F2) have an average error of 15% revealing that the rejected points of the contour were excessive.

### 4.4. Peaches with Occlusion Ratio from 33% to 66%

Figure 13 shows a sample image of a paraguayo peach with an occlusion of 34.5%. In a general analysis of partially occluded (from 34 to 66%) fruit pieces, the average error in the number of different pixels between a manually-labeled and automatically-segmented image was 12.2% with minimum and maximum values of 7.4% and 20.5% respectively.

Table 6 summarizes the average relative error in the estimate of the diameter of the fruit. In the case of a real segmented image (Figure 13-IS) the MD relative error was slightly higher (15.5%) than the MAL-F1 and MAL-F2 relative errors that were very similar.

### 4.5. Peaches with Occlusion Ratio Higher than 66%

Figure 14 shows a sample image of a paraguayo peach with a heavy occlusion of 77.9%. In a general analysis of heavily partially occluded (from 66%) fruit pieces, the average error in the number of different pixels between a manually-labeled and automatically-segmented image was 23.0% with minimum and maximum values of 13.7% and 26.1% respectively.

Table 7 summarizes the average relative error in the estimate of the diameter of the fruit. In the case of a real segmented image (Figure 14-IS) the MD and MAL-F1 relative errors were very high (above 80%) whereas the than the MAL-F2 was lower but also very bad (48.9%) for an estimator.

## 5. Conclusions

This work proposes the application of linear color models in the RGB vector color space to detect red paraguayo peaches in images from orchards taken under natural light. The proximity to the different linear color models is the criteria used to individually classify the pixels of the images and there is no need to define additional threshold or radial proximity distances in the linear color models. With this approach, the average error obtained when measuring the area of individual fruits by comparing the manual labeling and the automatic segmentation procedure was 11.6%. Specifically, in the case of bright lighting the average error in the estimate of the area (compared to a manual segmentation) was 19.7%, 8.2% in the case of low illumination, 8.6% in the case of occlusion of less than 33%, 12.2% for occlusion between 34 and 66%, and 23% in the case of occlusion higher than 66%. These results showed that there is a correlation between the percentage of occlusion and the accuracy of the segmentation performed.

Additionally, a procedure to estimate the diameter of one fruit was proposed. The overall conclusion is that the diameter of the fruit computed by measuring the major distance between pixels that define the contour of a non-occluded fruit in one image is in general very similar to the major axis length of a fitted ellipse. However, by applying a threshold level to the residual obtained in the non-linear fit, some pixels from this contour can be discarded in a second fit to obtain a new estimate of the diameter. Then, if both major axis lengths are similar, the fruit is not occluded and both values can be used as a diameter estimate; if the major axis length is longer when avoiding some pixels of the contour then the occlusion will be up to 33% and the result of the second fit must be discarded; and if the major axis length is shorter when avoiding some pixels of the contour then the occlusion will be higher than 66% and no diameter estimate can be performed.

In the future, efforts will be concentrated on defining the linear color models automatically and analyzing the application of the linear color models to other fresh fruits.

## Figures and Tables

**Table 1.** Description of the linear color models used (plotted also in Figure 5).

***Sub-Image***	***Sample***	***Label***	***Object***	***Linear Color Model Description***
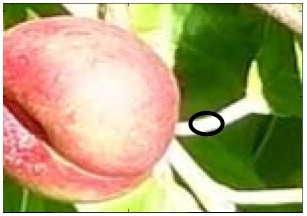	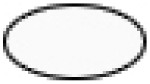	B1	Branch	Whitish branch (brightly illuminated and color saturated)
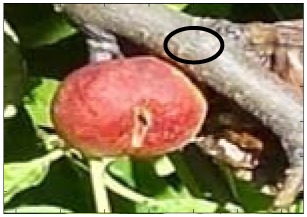	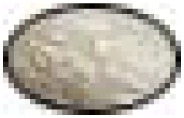	B2	Branch	Branch
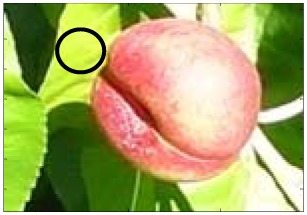	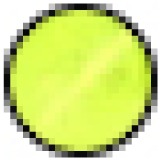	L1	Leaf	Yellowish leaf
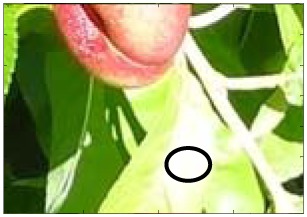	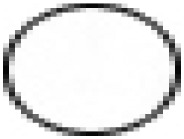	L2	Leaf	Whitish leaf
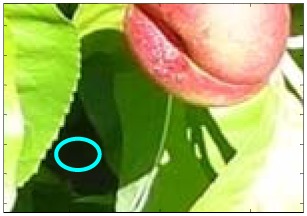	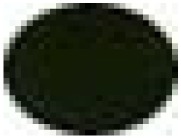	L3	Leaf	Dark (shaded) leaf
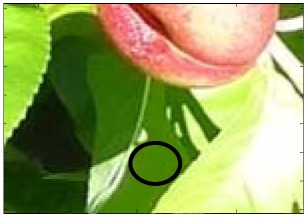	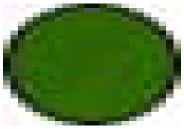	L4	Leaf	Leaf
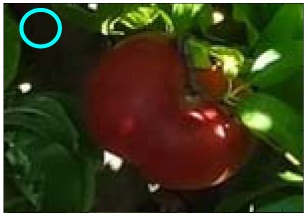	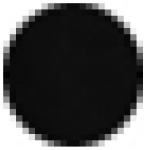	L5	Leaf	Dark area
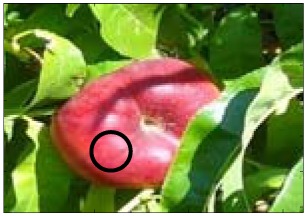	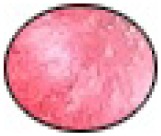	P1	Peach	Brightly illuminated peach
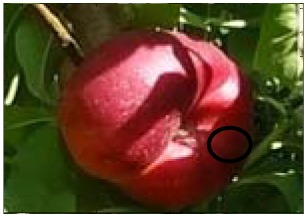	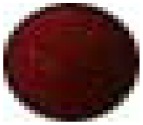	P2	Peach	Dark peach
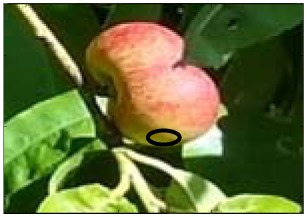	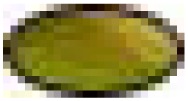	P3	Peach	Yellowish peach
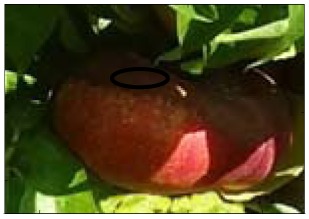	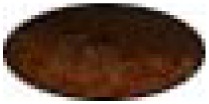	P4	Peach	Brownish peach

**Table 2.** Peach size estimate in the case shown in Figure 7.

**Analysis**	**Image**	**Parameter**	**Value (Pixels)**
Manual	LS	Maximum Distance (MD)	237
		Major Axis Length (MAL)	249
Automatic	IS	Maximum Distance (MD)	225
		Major Axis Length (MAL)	247

**Table 3.** Relative error in the diameter estimate: bright illumination case.

**Image**	**Diameter Measurement Method**	**Error (%) Relative to MD in Image LS**		

		**Min**	**Average**	**Max**
LS	MAL-F2	1.0	2.7	5.1
	MD	2.4	6.4	12.1
IS	MAL-F1	0.7	3.5	8.2
	MAL-F2	0.8	3.6	8.2

**Table 4.** Relative error in the estimate of fruit diameter: low illumination case.

**Image**	**Diameter Measurement Method**	**Error (%) Relative to MD in Image LS**		

		**Min**	**Average**	**Max**
LS	MAL-F2	0.3	1.6	3.0
	MD	0.5	6.2	27.3
IS	MAL-F1	1.6	5.6	23.0
	MAL-F2	0.2	8.0	26.5

**Table 5.** Relative error in the estimate of fruit diameter: occlusion ratio lower than 33%.

**Image**	**Diameter Measurement Method**	**Error (%) Relative to MD in Image LS**		

		**Min**	**Average**	**Max**
LS	MAL-F2	0.1	1.2	3.8
	MD	0.5	5.8	16
IS	MAL-F1	0.8	5.6	12.2
	MAL-F2	0.2	15.0	60.5

**Table 6.** Relative error in the estimate of fruit diameter: occlusion ratio from 33% to 66%.

**Image**	**Diameter Measurement Method**	**Error (%) Relative to MD in image LS**		

		**Min**	**Average**	**Max**
LS	MAL-F2	0.4	1.8	4.1
	MD	2.0	15.5	35.2
IS	MAL-F1	1.1	10.5	24.2
	MAL-F2	1.8	12.8	23.9

**Table 7.** Relative error in the estimate of fruit diameter: occlusion ratio higher than 66%.

**Image**	**Diameter Measurement Method**	**Error (%) relative to MD in Image LS**		

		**Min**	**Average**	**Max**
LS	MAL-F2	1.0	3.7	5.4
	MD	80.5	96.7	128.8
IS	MAL-F1	58.5	89.0	124.9
	MAL-F2	30.7	48.9	75.6
